# Associations of sympathetic and parasympathetic activity in job stress and burnout: A systematic review

**DOI:** 10.1371/journal.pone.0205741

**Published:** 2018-10-18

**Authors:** P. C. de Looff, L. J. M. Cornet, P. J. C. M. Embregts, H. L. I. Nijman, H. C. M. Didden

**Affiliations:** 1 Behavioural Science Institute, Radboud University, Nijmegen, The Netherlands; 2 Wier, Specialized and Forensic Care, Fivoor, Den Dolder, The Netherlands; 3 Expertcentre “De Borg”, Den Dolder, The Netherlands; 4 Psychology of Conflict, Risk and Safety, University of Twente, Enschede, The Netherlands; 5 Department of Tranzo, Tilburg School of Social and Behavioral Sciences, Tilburg University, Tilburg, The Netherlands; 6 Trajectum, Specialized and Forensic Care, Zwolle, The Netherlands; Universita degli Studi di Pisa, ITALY

## Abstract

This systematic review examines the relationship between sympathetic and parasympathetic activity on the one hand and job stress and burnout on the other, and is registered at PROSPERO under CRD42016035918. Background: Previous research has shown that prolonged job stress may lead to burnout, and that differences in heart rate variability are apparent in people who have heightened job stress. Aims: In this systematic review, the associations between job stress or burnout and heart rate (variability) or skin conductance are studied. Besides, it was investigated which–if any–guidelines are available for ambulatory assessment and reporting of the results. Methods: We extracted data from relevant databases following the PRESS checklist and contacted authors for additional resources. Participants included the employed adult population comparing validated job stress and burnout questionnaires examining heart rate and electrodermal activity. Synthesis followed the PRISMA guidelines of reporting systematic reviews. Results: The results showed a positive association between job stress and heart rate, and a negative association between job stress and heart rate variability measures. No definite conclusion could be drawn with regard to burnout and psychophysiological measures. No studies on electrodermal activity could be included based on the inclusion criteria. Conclusions: High levels of job stress are associated with an increased heart rate, and decreased heart rate variability measures. Recommendations for ambulatory assessment and reporting (STROBE) are discussed in light of the findings.

## 1. Introduction

Job stress can be divided into momentary and prolonged stress. Particularly, prolonged job stress may lead to burnout [1–3], which has substantial negative socioeconomic consequences. Traditionally, job stress and burnout are measured with self-report questionnaires that are often based on a specific theoretical model. For instance, the Effort Reward Model defines job stress as an imbalance between the efforts and rewards on the job [4] while the often used Maslach Burnout Inventory defines burnout as a combination of exhaustion, cynicism and decreased personal accomplishment [5]. In addition to cognitive, emotional, and behavioral effects, prolonged job stress has detrimental effects on cardiovascular functioning of human beings [6], which is primarily controlled by the autonomic nervous system [7]. In this systematic review, we focus on the association between job stress and burnout on heart rate (variability) and skin conductance.

The human body maintains balance of key regulatory functions such as temperature, metabolism and heart rate through the autonomic nervous system (ANS) [7]. This system consists of two major branches; a sympathetic nervous system (SNS) and a parasympathetic nervous system (PNS). Both branches play a crucial role in the immediate stress regulatory response of the body [8]. The SNS facilitates behavioral activation in response to perceived threat (fight/flight response), resulting in, for instance, increased heart rate and sweat production. The PNS, on the other hand, facilitates homeostasis of the body (rest/digest situation), resulting in, for instance, reduced heart rate [9].

The human body maintains balanced through the ANS by *efferent* (neurons that carry impulses outward from the brain and spinal cord to an effector such as organs) and *afferent* (neurons that carry peripheral impulses to the brain or spinal cord) nerves [10]. In the face of a stressor, which can be both physical and nonphysical [8], a range of complex reverberating systems is activated to deal with the stressor [11] such as higher order brain processes, coordination of blood flow, heart rate, breathing rate, release of hormones, and activation of muscle fiber to react to the stressor [11]. In terms of time, the parasympathetic effects on heart rate act within milliseconds, while the sympathetic effects on heart rate and skin conductance act in seconds [12,13].

Heart rate is primarily controlled by tonic vagal (parasympathetic) inhibition of the heart. The vagus nerve primarily acts on the sinoatrial node (responsible for pace of the heart rate), while the SNS primarily acts on the atrioventricular node (responsible for the force of contraction). The inhibitory effects of the vagus nerve result in slower heart rate while disinhibitory effects increase heart rate [8,14].

The Polyvagal theory explains the flow from body to brain from an evolutionary perspective. According to this evolutionary perspective, the vagus nerves (which is the tenth cranial nerve) plays a key role in the ANS. The myelinated branch of the vagus nerve is assumed to be the most sophisticated part and to control SNS activity [15,16]. Lower PNS activity therefore indicates less control of the myelinated branch, resulting in less inhibition of the fight/flight characteristic of the SNS. Although the Polyvagal perspective on PNS and SNS control is under debate [17,18], there is a relatively broad consensus that especially dysregulation of the PNS underlie emotional and behavioral problems. In line with this, in chronic stressed participants, a hypoactive PNS is usually observed with disinhibition of sympathoexcitatory circuits with the phenomena of increased HR and increased blood pressure. As a result of prolonged energy mobilization different phenomena occur such as allostatic load [19], irritability [11] or a feeling of exhaustion [10]. Feeling stressed or burned out from work is the result of a complex interplay between the brain, spinal cord, and ANS in which the interoceptive afferent neural system is responsible for becoming aware of the physiological state of the body [10], and is mainly caused by the afferent function (80%) of the vagus nerve [20].

According to Vrijkotte et al. [21], the detrimental effects of job stress are the result of sympathetic activation in combination with parasympathetic withdrawal. In the following paragraphs we will first focus on the stress response in relation to job stress and burnout in order to provide the reader with some common conceptualizations of job stress and burnout. Following this, we will discuss heart rate and skin conductance measures that were analyzed in this review.

According to Boucsein [12], stress “can be defined as a state of high general arousal and negatively tuned but unspecific emotion, which appears as a consequence of stressors (i.e., stress-inducing stimuli or situations) acting upon individuals. Stressors can be defined as subjective and/or objective challenges exceeding a critical level with respect to intensity and/or duration” (p.381), which is in accordance with the theory of cognitive appraisal [22]. A stress reaction can be described in cognitive, emotional and physiological responses [23]. The cognitive and emotional responses traditionally have been measured with self-report questionnaires, whereas the physiological response can be quantified through both biomedical (e.g., blood, urine, and saliva) and autonomic nervous system markers (blood pressure, respiration rate, heart rate, and skin conductance) [12,23,24].

More specific cases of stress, i.e. job stress and burnout have been described in association with autonomic nervous system markers. These markers have increasingly been the subject of research over the past decades [25]. Technological advances enable users to monitor these markers over a prolonged period of time using ambulatory devices. Both autonomic nervous system markers of heart rate and Electrodermal Activity (EDA; often recorded as skin conductance, or skin resistance p. 2) [12] have been shown to be related to job stress and burnout, and are the primary focus of the current systematic review.

### 1.1 Theoretical models on job stress and burnout

Burnout has been proposed as one possible outcome of prolonged job stress since the 1980’s [26]. Job stress and burnout are mostly measured with self-report questionnaires. The two most often used models to assess job stress are the demand-control model [27] (and the more recent demand-control-*support* model) [28] and the effort-reward imbalance model [4,29]. The first model distinguishes between demands and control on the job. Demands are measured in terms of time, quantity, and mental variables on the job whereas control is measured as the amount of decision latitude, growth possibilities, and the amount of creativity one is able to put in one’s work [30]. There is a reciprocal relationship between demands and control, where an imbalance towards high demands/low control is used to describe job stress. The second theoretical model distinguishes effort and reward on the job. Efforts are measured with variables such as demands, workload, and work pressure whereas reward is measured in terms of monetary incentives, self-esteem, and career opportunities [4]. An imbalance between the two is referred to as high cost and low gain. Siegrist et al. [4] put it as follows: “in the long run, the imbalance between high effort and low reward at work increases illness susceptibility as a result of continued strain reactions” (p. 1485).

Next to job stress, burnout is also defined in various theoretical models. A well-known and influential model of Maslach [31] characterizes burnout as a feeling of exhaustion and depersonalization, with low levels of personal accomplishment. Exhaustion includes feelings of being used up or emotionally drained by one’s work [32]. Depersonalization is characterized by feelings of callousness towards other people, while low personal accomplishment is described in terms of the perceived impact of one’s work. Considering that burnout is a possible severe reaction to (prolonged) job stress, we hypothesize that if burnout is the result of job stress, the effect of burnout on the autonomic nervous system might have an even stronger influence than the effect of job stress alone.

In the following paragraphs we will first discuss some common heart rate measures followed by the skin conductance measures that were analyzed in this systematic review.

### 1.2 Physiological measures of job stress and burnout

HR can be analyzed both in terms of beats per minute and in terms of the inter-beat interval (IBI). The mathematical analysis of HRV is based on the variation of the IBI interval [33], and can be divided in the amount of parasympathetic or mixed (both parasympathetic and sympathetic) activity that is reflected [9,13,34–36]. HRV can be calculated in both *time domains* and *frequency domains* [37] (and nonlinear analysis, but this was not included in the current review). Three *time domain* measures are used in the current review and are based on the variation in peak to peak interval. The standard deviation of these peak-to-peak intervals is also referred to as the standard deviation of normal-to-normal intervals (SDNN), which is a measure of overall HRV [37]. In addition, the percentage of adjacent cycles greater than 50ms apart (PNN50) and the root mean square of successive differences (RMSSD) are used. A *Frequency domain* measure is also included. These measures are based on the analysis of peak-to-peak (RR) interval sequences [33] as well. The frequency components can be calculated as the distribution of power (i.e. variance of the peak-to-peak interval) as a function of frequency. The high frequency power is thought to primarily reflect parasympathetic activity while the other measures reflect mixed activity [35]. Only the HF component is included in the current review (0.15–0.4 Hz).

The measures in the current review are predominantly parasympathetic (PNN50, RMSDD, HF) or sympathetic (EDA measures) in nature. Besides HR, a second mixed measure was included (SDNN) that is traditionally viewed as HRV [37]. SDNN is sometimes considered as the total measure of autonomic nervous system activity (see for an overview, p. 1813) [35].

There is considerable heterogeneity in methodology and measurement that might influence the recordings of HR. For instance, studies report rest measures [38,39], 24 hour recordings [40,41], HR measures during controlled breathing [39] or during a specific task [42]. In addition, ECG (electrocardiogram) devices [30,43,44], blood pressure devices [45,46] or ambulatory PPG (photoplethysmography) sensors [47] are used. Studies report on measures during work [48], leisure time [21], rest [49] or at night [46]. Studies report on untransformed values [45,50], linear transformations [41,51] or both [49]. Bivariate measures are sometimes reported [13,39] or studies report on adjusted models [6,52]. These choices might seriously influence the results that are reported. Moreover, there is significant diurnal variation in both HR [53,54] and EDA [55] which makes the comparability between studies that use different lengths of recording or different time intervals challenging.

EDA is relevant with respect to skin conductance or skin potential [9]. It is one of the most sensitive markers of arousal [12,56], and solely the result of the sympathetic activation of the autonomic nervous system [12]. Although EDA has been studied extensively in experimental research on (among others) anxiety, stress, depression, and personality, it has not often been reported as a marker specifically in association with job stress. This might be due in part to the equipment that was needed to measure EDA in the workplace (e.g., multiple sensors on the fingers and/or hand palm). Recent technological advances make it easier and less intrusive to measure EDA [57,58]. EDA has both tonic (level) and phasic (responses) components. The typical form of a response is well described [12], and several parameters can be extracted such as the height, rising time, area under the curve, or decay time of a response. For this review we will focus on the skin conductance level (SCL), the number of non-specific skin conductance responses per minute (ns.SCR) and the height (amplitude) of the non-specific responses (SCR.amp), as these have been associated most with emotional load in job-related EDA research (pp. 460–462) [12]. Boucsein [12] reported results from a few studies on the association between EDA and job stress. There is a tendency of increased SCL, ns.SCR, and SCR.amp with increased strain and stress. Therefore, these markers will also be addressed in this systematic review.

A recent systematic review on job stress and HRV concluded that “stress at work is generally associated with increased risk of disease and worsened health profiles as indicated by decreases in vagally-mediated HRV.” (p. 1814) [35]. Where these authors looked at both mixed and vagally (parasympathetic) mediated HRV measures in association with job stress, we will focus on these measures (RMSSD, PNN50, HF, HR, SDNN), as well as on EDA in association with both job stress and burnout. Moreover, we will also assess the direction of the associations, even if the effect is non-significant [35]. In addition, the former review analyzed both total as well as subscales of job stress, while this review will solely rely on total validated scales for both job stress and burnout. Only the full scales of the questionnaires were used as the current review focused on the association between both EDA and HR(V) with job stress and burnout. This served as a means to compare the results on ‘job stress’, and is an important distinction with the Jarczok [35] review that investigated the more general ‘workplace stressors’ as both full and subscales were considered [35]. For instance, Jarczok et al., [35] reported that need for control significantly decreased HF in the Hanson [59] study. Although need for control is a subscale of the job stress questionnaire, it is not necessarily considered to be job stress. People can experience heightened demands, but if control is not decreased there is not a ‘pure’ association with job stress as a full scale. The results are thus only applicable to workplace stressors, but not job stress. As the current review compared job stress to burnout, only full scales were considered to compare the job stress-burnout association instead of making comparisons between depersonalization and demands for instance.

This systematic review could therefore be considered, at least in part, as both a replication, update and an extension of their previous work. In sum, we want to know what the association is between job stress/burnout and HR(V)/EDA, which parameters might prove useful, and which recording periods are favorable over others to analyze.

Based on the outcomes of the previous review, three specific hypotheses are formulated. First, it is expected that there are positive associations between job stress/burnout and HR and EDA. Second, negative associations between job stress/burnout and HRV are expected. Third, the association between burnout and HR(V)/EDA is stronger than the association between job stress and HR(V)/EDA as burnout is a possible result of severe, enduring and prolonged job stress. Participants will include the employed adult population comparing validated job stress and burnout questionnaires examining heart rate and electrodermal activity. In addition, the timeframes that are used to assess the physiological measures vary considerably, therefore this review also aims to provide some guidelines of measurement and reporting.

## 2. Method

### 2.1 Literature search and screening criteria

The literature search focused on the relationship between job stress and burnout on the one hand, and EDA, and HR(V) on the other. The review protocol was registered in the international prospective register of systematic reviews (PROSPERO) with ID number CRD42016035918. We used the Preferred Reporting Items for Systematic reviews and meta-analysis (PRISMA) to guide the reporting on the systematic review [60]. The databases that were searched for this systematic review were PsychINFO, Medline, Embase, and Web of Science. For this, we used the subscription of the Radboud University in Nijmegen, the Netherlands. The search engines, and accordingly, the search terms of the databases differ; for this reason we included the search terms in S1 Appendix which included search terms on HR, HRV, EDA, burnout and job stress. The search terms were peer-reviewed by three librarians using the Peer Review of Electronic Search Strategies (PRESS) checklist [61], which resulted in some additional suggestions, the narrowing of search terms and addition of relevant keywords. As the task force guidelines for HR measurement were published in 1996 [37] and needed some time to be adopted, it was decided to include articles from 2000 upwards. Zotero (v. 5.0.52) and Refworks were used to process the references.

**Fig 1:**
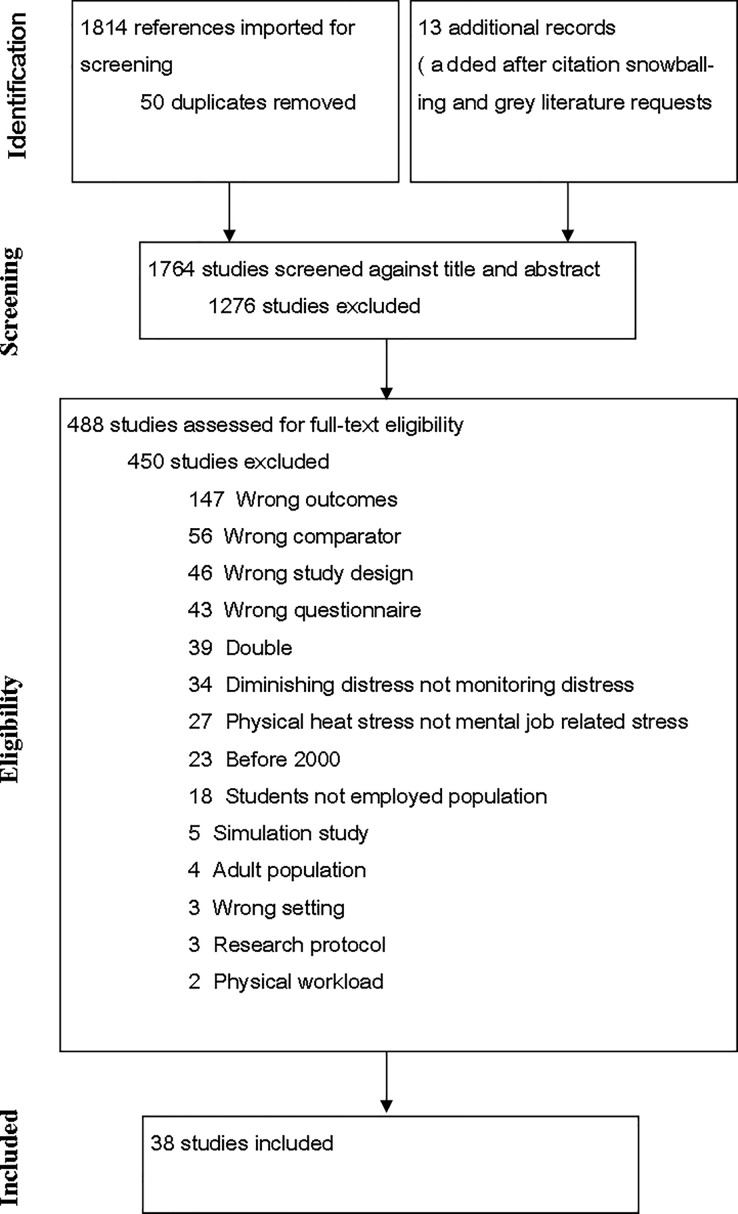
PRISMA flow chart of the systematic review process.

The final search was conducted on December 23^rd^ 2016. The final searches yielded a total number of 1,814 studies. Besides citation snowballing, every included study author (only the corresponding authors) was contacted to ask if they were aware of any further or so-called ‘grey’ literature, which yielded an additional 13 studies. In the end, we included 38 articles (see Fig 1).

### 2.2. Study selection

The following inclusion criteria were applied:

1. The studies focused on the employed adult population and should concern job stress or burnout related measures, as predictor variables and HR(V) or EDA as outcome variables.
2. The studies focused on baseline, workdays, leisure time or combinations of those.
3. The studies included a validated subjective measure of job stress or burnout.
4. The included studies were English articles published between 2000 and 2016.

We had no further requirements as far as the study design or participants were concerned. Comparisons were made based on validated questionnaires. The HR(V) and EDA measures were divided in separate outcomes for rest, task, workday, leisure, sleep time or combinations of the entire period. We performed an initial screening with three (PCdL, HN, PE) reviewers to establish if the inclusion and exclusion criteria were transparent. For this purpose, two sets of 50 randomly sampled articles were used to establish interrater agreement. This initial screening resulted in 85% interrater agreement on the title and abstract screening, and consensus on transparency of the inclusion and exclusion criteria.

We set out to perform a meta-analysis as this would allow for explaining heterogeneity in effect sizes through moderator analysis [62, pp. 198–228]. However, most of the articles did not report on bivariate correlations or means. A meta-analysis therefore would have resulted in a limited number of processed articles. We could have opted for the use of partial correlations, but there is debate on the use of them [63], even if the results of the bivariate and partial analyses are reported separately. Because we were unable to perform moderator analyses in a meta-analysis we decided to do an exploratory analysis on sample size, sex differences and age as these are expected to moderate the association between psychophysiology and job stress/ burnout.

### 2.3 Coding and reporting

Two authors double coded the effect directions and significance levels of the included articles (performed by PCdL and LJMC), and the interrater agreement was 88%. After a consensus meeting, both authors agreed on 100% of directions and significant associations. In addition, the articles were coded on the HR(V) or EDA outcomes, and the period of analysis, the time of the HR(V) and EDA measurement, the applied stress model (job stress, burnout), the time of stress measures, and the cut-off used for making subgroups of the participants. Furthermore, several other measures were coded (21 of 38 articles were double coded by (PE, HN, RD)). The risk of bias assessment for all articles are presented in S3 Appendix. In order to avoid simply looking at *p*-values (vote counting), we also looked at the direction of the comparisons. A positive direction means that higher levels of job stress and burnout were associated with higher levels of HR(V) and EDA. In case the articles reported mean differences or correlation coefficients these were used to describe the found effects. If no tables or information was available the wording of the authors was coded. For instance, Uusitalo [51] only reported all significant correlations in a table and concluded in the text that “the pure vagal time-domain index RMSSD was the only HRV measure which correlated with ER-imbalance” (p. 835). Since that was the only significant effect, it was assumed that the other investigated measures in the study, that is both HF and SDNN, were non-significant. These measures were therefore set to “no effect” and significance level as “not reported” because we also did not have information on the direction of the association. If tables were available we extracted the direction of the association from the tables, if no significance test was applied to the subgroups in those tables we reported that the significance was n.r. (not reported). All significance levels were set at *p* < .05. All available data is included with the article to both enhance reproducibility and compliance with the PLOS policy.

## 3. Results

### 3.1 Study characteristics

We included 38 articles which reported on 119 outcome measures (Table 1). All reported outcomes in these 38 articles turned out to be HR(V) measures; No EDA studies met the inclusion criteria. In the initial full text review, we did identify 4 EDA studies that were possibly eligible, but they were excluded because of the use of a non-validated job stress questionnaire (2 studies) or because it was a simulation study (1 study), or a real-life stress exposure (1 study).

**Table 1:** Descriptive characteristics of the articles.

Nr of articles included	38
Nr of reported outcomes	119
Sample size range	17–9924
Articles age range^a^	26.9–51.2
Articles with only female samples^b^	6
Articles with only male samples	11
Articles with mixed samples^c^	18
Articles with no report on sex distribution	3
Articles reported on burnout	9
Articles reported on ERI	7
Articles reported on JDC	18
Articles reported on ERI and JDC	3
Articles reported on Organizational Injustice	1

^a^Seven articles did not report on age

^b^It should be noted that the article by Hintsanen et al. [39] reported on men and women separately. Both men and women were analyzed separately

^c^The remaining 18 articles ranged from 8–95% as far as the inclusion of women was concerned.

Nineteen of 38 study authors responded to requests of grey literature or additional information. For four authors we were unable to retrieve a valid email address.

### 3.2 Association between both burnout and job stress with (para)sympathetic measures

The first hypothesis states that there is a positive association between HR and job stress/burnout. The associations between HR/SDNN and job stress/burnout are summarized by recording period in S2 Appendix. There were thirty-five reported outcomes on HR, of which 32 outcomes are reported in the Appendix. As can be seen in S2 Appendix, the majority of articles on HR and job stress/burnout found positive associations. The three non-reported outcomes were difficult to categorize (see Table 2). First, Poorabdian et al. [64] reported on an omnibus Chi-square test of which it was unclear at which time point the measures were taken. However, the direction of the association was significantly positive. Second, only one reported outcome for the Moya-Albiol et al. [48] study was significantly negative while one outcome was mixed. The study reported a significant negative association caused by measures in the middle of the workday. Third, Borchini et al. [40] reported on a non-significant positive relationship during a period of leisure and night. Therefore, in sum, twelve reported outcomes were significantly positive effects. The ratio of significant effects is in favor of a positive association (12 positive effects vs 2 negative effects; 12:2). Finally, seventeen outcomes reported on SDNN, of which three reported outcomes were significantly negative, and one was significantly positive.

**Table 2:** Codesheet from the included articles.

Study	HRV	Effect direction (+ = positive, - = negative)	Significant at the p < .05 level? (y/n)	Time HR assessment	Stress Measure	Study design; Year; Country	Sample Description	N analysis; N reported	Age Mean; Range; %Female; Stress division	Remarks
(Bishop et al., 2003)[65]	HR_w	+	y	every 30 min during a workday	JDC	C; -; Singapore	Singapore Police Officers	108; 118	26.9; 19–50; -; SD	
(Borchini et al., 2014)[40] and (R. Borchini, personal communication, March 1st, 2017)	HF_l	-	n	2 24 hour days continuous	JDC and ERI both used to identify high strain	L; 2010; Italy	CVD susceptible nurses	36	38.1; -; 83.3; E/R D/C	
	HF_wln	-	y							
	PNN50_l	-	n							
	PNN50_wln	-	n							
	RMSSD_lln	-	n							
	RMSSD_wln	-	n							
	SDNN_lln	-	n							
	SDNN_wln	-	y							
	HR_lln	+	n							
	HR_wln	+	n							
(Butterbaugh et al., 2003)[66]	HR_r	ne	n	-	JDC	C; -; -	Newly employed female nurses	58	-; -; 100; D/C	
(Chandola et al., 2008)[67]	HF_r	-	y	5 min RR was used	JDC	L; 1985–2004; Great Britain	Civil servants	3290	-; 35–56; -; Mdn	
	SDNN_r	-	y							
(Clays et al., 2011)[43]	HF_wln	-	n	24 hours including workday, HRV measures are based on 24 h	JDC	C; 1976–1978; Belgium	Healthy male factory workers	653; 770	47; 40–55; 0; Sum	
	PNN50_wln	-	n							
	SDNN_wln	-	n							
	HR_wln	+	y							
(Collins et al., 2005)[68]	HF_w(l)ln	-	y	48 hours, including work and rest days	JDC	C; -; United States	Healthy employed day shift working men from a community health plan and N = 6 from a stress reduction program	34; 36	45; 35–59; 0; Tri	
	HF_w	-	n							
	SDNN_w(l)ln	+	n							
	SDNN_w	-	n							
	HR_w(l)ln	+	n							
	HR_w	+	y							
(van Doornen et al., 2009)[69]	HF_l	-	n	24 hour workday	Burnout (Maslach)	C; -; Netherlands	Male managers of a Dutch telecommunications company	88	43.3; -; 0; HLC	
	HF_n	-	n							
	HF_w	-	n							
	HR_l	-	n							
	HR_n	+	n							
	HR_w	+	n							
(Ekstedt et al., 2004)[70]	HR_n	+	y	24 hour, but HR measured at rest before awaking at 7 am +/- 1 hour	Burnout (Shirom Melamed)	C; -; Sweden	Employees of IT company	24	30.5; 24–43; 58.3; HLC	The significant effect must be interpreted with caution, it is part of a multiple regression analysis and burnout group is entered as a dummy.
(Eller et al., 2011)[38]	HF_r	-	n	18 hour ECG starting on a workday in both 2006 and 2008, but only 15 min logtransformed seated rest in analysis	ERI	L; 2006–2008; Denmark	White collar workers in the public administration males	61	51.2; -; 0; E/R	
	HF_r	-	y							
	HR_r	+	y							
	HR_r	+	y							
(Eriksson et al., 2016)[71]	HR_r	+	y	5–10 min resting period	JDC	C; 2001–2004; Sweden	Working population	1552	46; 24–71; 52; D/C	
(Fauvel et al., 2001)[72]	HR_r	ne	n	Measured during 15 minutes of seated rest	JDC	C; 1995–2001*; France	workers of a chemical company	281	37.3;18–55; 8; HL	Top 20% was considered a high strain group
	HR_t	+	n							
	HF_r	+	n							
	HF_t	+	n							
(Hamer et al., 2006)[45]	HR_r	+	n	10 min BP (measured last 5 min of a 10 min resting period)	ERI	C; 2003–2004; Great Britain	full time employed men	92	33.1; -; 0; E/R	
	HR_t	-	y	8 min BP measured during a role playing and mirror tracing tasks						
(Hanson et al., 2001)[59]	HF_w(l)	-	n	During a working day, but for the office clerks the measurements continued into the evening (until 21.30)	ERI	C; -; Netherlands	Health professionals and office clerks	70	36.3; -; 44; E/R	Only seated periods were analyzed
(Henning et al., 2014)[47]	RMSSD_n	ne	n	24 hour Amb measurement, but the unit of analysis is data between 2 am and 4 am	Burnout (Copenhagen Burnout Inventory)	L; -; New Zealand	junior doctors	17	-; 20-?; 65; Mean	In the conclusion it states that there were no doctors with burnout, so there is actually nothing to compare
	RMSSD_wl	ne	n							
(Hernández-Gaytan et al., 2013) [73]	HF_w	-	n	24 hour ECG workday, 8 hour shift and 16 hour on call time	JDC	C; 2007–2008; Mexico	resident doctors	54	-; 23–36; 33; Mdn	
	SDNN_w	-	n							
(Herr et al., 2015)[41] and (R. Herr, personal communication, February 22nd, 2017)	HF_n	+	y	24 hour ECG	OI	C; 2007; Germany	White collar workers	179	46.4; -; 0; Sum	Sum is total OI scale
	HF_wln	+	n							
	RMSSD_n	+	y							
	RMSSD_wln	+	n							
	SDNN_n	+	y							
	SDNN_wln	+	n							
	HF_n	-	n				Blue collar workers	222	44.3; -; 0; Sum	
	HF_wln	-	n							
	RMSSD_n	-	n							
	RMSSD_wln	-	n							
	SDNN_n	-	n							
	SDNN_wln	-	n							
(Hintsanen et al., 2007)[39]	HR_r	-	n	3 min controlled breathing	ERI	C; 2001–2002; Finland	Employed people working full time males	406	32.2; 24–39; 0; E/R	
	HF_r	+	n							
	PNN50_r	+	n							
	RMSSD_r	+	n							
	HR_r	+	n					457	32.3; 24–39; 100; E/R	
	HF_r	-	n							
	PNN50_r	-	y							
	RMSSD_r	-	y							
(Jarczok et al., 2016)[61]	RMSSD_n	-	y	24 hour workday continuous	ERI	C; 2010–2012; Germany	Mannheim Industrial Cohort (MICS)	9924; 9937	41.9; 18–65; 19; E/R	
	RMSSD_wl	-	y							
(Johnston et al., 2016)[74]	HR_w	+	n	2 12 hour workdays	JDC and ERI	C; -; Great Britain	Qualified nurses in a general hospital on medical and surgical wards	100	36.4; -; 93; Sum	
(Jönsson et al., 2015)[3] and (P. Jönsson, personal communication, February 16 th, 2017)	HR_t	-	n	1 hour ECG during task and recovery with a baseline reading	Burnout (Shirom Melamed)	Lab; -; -	Employed population with N = 14 Former ED (Burnout) patients, n = 17 pre ED stage workers and n = 20 controls	51	48.7; 33–61; 51; HLC	
	HF_t	+	n							
(Kang et al., 2004)[49]	HF_r	-	n	5 minutes in the morning	JDC	C; 2003; South-Korea	Male shipyard workers	169	46.7; 41-?; 0; D/C	
	SDNN_r	-	n							
(Karhula et al., 2014)[75]	HR_n	-	n	3 non consecutive 24 hour days including a morning shift, night shift and recovery day. Data used for analysis was at least 4h of sleep after which the 30 min segment with the lowest heart rate was used for analysis.	JDC	C; 2008; Finland	Female nurses	95	47.2; 31–59; 100; mdn and mean	mdn and mean (to get a greater contrast)
	HF_n	ne	n							
	RMSSD_n	ne	n							
(Kotov and Revina, 2012)[50]	HF_w	-	y	8 hour workday	Burnout (Boiko)	C; -; Russia	First-aid doctors	44; 84	-; 26–65; 56; HLC	Both coping strategy groups show a negative effect
	PNN50_w	+	n							The effect is negative for a task-oriented coping strategy. The effect is positive for an emotion oriented strategy.
	RMSSD_w	-	n							The effect is negative for a task-oriented coping strategy. The effect is positive for an emotion oriented strategy.
	SDNN_w	-	n							Burnout (Alarm stage vs no Burnout). Article uses coping strategies as comparator. The effect is negative for a task-oriented coping strategy. The effect is positive for an emotion oriented strategy.
(Lee et al., 2010)[76]	HF_r	+	n	Measured 3 times in each subject after completion of 1 day, 1 night and 1 eveningshift in a 5 min rest period after 5 min of rest.	JDC	C; -; South-Korea	Employees of consumer goods company	56; 140	29.1; 25–44; 0; D/C	
(Lennartsson et al., 2016)[52]	HF_r	-	y	5 minutes in the morning in supine position	Burnout (Shilom-Melamed)	Lab; -; Sweden	Employed, working and on sick leave burnout patients, non-clinincal burnout subjects and healthy controls	161	40; 20–65; 60; HLC/HL	The effects are only significant in men.
	RMSSD_r	-	y							
	SDNN_r	-	y							
(Loerbroks et al., 2010)[30]	RMSSD_l	ne	n	24 hour wln measure	ERI	C; 2003–2004; Germany	Employees from an airplane manufacturer	591; 657	41.6; 17–65; 12; E/R	Some positive, some negative, only age group 35–44 negative significant effect
	RMSSD_l	ne	n		JDC					
	RMSSD_n	ne	n		ERI					
	RMSSD_n	ne	n		JDC					
	RMSSD_w	ne	n		ERI					
	RMSSD_w	ne	n		JDC					
(Morgan et al., 2002)[32]	HF_r	+	y	10 min in supine position	Burnout (Maslach)	C; -; United States	Soldiers	41	-; -; -; HL	
(Moya-Albiol et al., 2010)[48]	HR_w	-/ne	y/n	3 times a day 30 min during seated rest	Burnout (Maslach)	C; -; Spain	Full- time school teachers	64; 80	42.8; -; 80; Mean	This effect was caused by HR at the middle of the day, which was significantly negatively correlated, the beginning of the day was positive, and the end of the day negatively related. Those were non-significant.
(Nomura et al., 2005)[77]	HR_r	+	n	After 5 min of rest measures were taken at rest	JDC	C; 2003; Japan	Employees from IT company	396; 437	30; 24–39; 0; D/C	20% highest were allocated to high job strain group
(Ohira et al., 2011)[42]	HR_r	+	y	During baseline and 2 learning tasks	JDC	Lab; -; Japan	Full time employed men	20	32.6; -; 0; D/C	D/C (but with median split in the sample)
	HF_r	+	n.r.							
	HF_t	+	n.r.							
(Poorabdian et al., 2013)[64]	HR_?	+	n.r.*	-	JDC	C; 2007–2009; Iran	Male personnel at a petrochemical plant	500	42.5; 22–64; 0; HL	* The authors presented a Chi-square. The percentage of people with the highest heart rate was highest in the high job strain group. (only the omnibus test is presented for all 12 categories)
(Rau, 2001)[44]	HR_n	ne	n.r.	Every 15 minutes over 24 hours during a normal working day	JDC	C; 1985-?; Sweden	Employed hypotensive (n = 74) and hypertensive (n = 75) men	81; 149	50.1; 35–55; 0; Mean	Mean (they use z scores). In the regression analysis, both control and support scales have a negative effect. The demand scale is not significant as it is not reported, the direction is therefore unclear.
	HR_w	ne	n.r.							
(Riese et al., 2004)[78]	HR_w(l)ln	ne	n	2 days, for 24 hours on a workday and one on a leisure day	JDC	L; 1997–1998; Netherlands	Healthy female nurses	159	35.9; 25–50; 100; Median	Median with a distinction of the four quadrants each year. High job strain year 1 and 2, yes or no. (results in 4 groups, y-y, n-n, y-n, n-y)
	RMSSD_w(l)ln	ne	n							
(Salavecz et al., 2010)[79]	HF_l	-	y	Measured over the working day	JDC	C; -; Hungary	Women working in Budapest	169	-; -; 100; -	They report on data after work
(Teisala et al., 2014)[80]	RMSSD_w	-	n	3 24 hour workdays, HRV measures are based on 24 h, not all participants three days. One day (n = 10, two days (n = 70), three days (n = 1).	Burnout (bergen)	C; -; -	Employed people	81	34; 26–40; 0; Mean	
(Uusitalo et al., 2011)[51]	HF_w	ne	n	2 36 hour workdays	ERI	C; -; Finland	Healthy hospital workers	19; 22	42; 24–57; 95; E/R	On day 2 it was significant, not on day 1
	RMSSD_w	-	y/n							
	SDNN_w	ne	n							
(van Amelsvoort et al., 2000)[44]	HR_n	-	n	24 hour workday	JDC	C; -; Netherlands	Shift workers and daytime workers as controls, working in the manufacturing industry, waste incinerator industry and hospitals	135; 155	30.8; 18–55; 19; D/C	For SDNN_n the contrast between high D, H control and low stress was significant)
	HR_w	+	y							
	HF_n	-	n							
	HF_w	-	n							
	SDNN_n	-	n							
	SDNN_w	+	n							
(Vrijkotte et al., 2000)[21]	HR_l	+	y	2 24 hour workdays and 1 24 h non workday	ERI	C; 1996–1997; Netherlands	White collar workers of a computer company	109	47.2; 37–57; 0; E/R	
	HR_n	+	n							
	HR_w	+	y							
	RMSSD_l	-	n							
	RMSSD_n	-	n							
	RMSSD_w	-	n							

JDC = Job demands control; ERI = Effort reward imbalance; OI = Organizational injustice; C = Cross-sectional; L = Longitudinal; Lab = Laboratory; HR = Heart rate; HF = High frequency; RMSSD = Root mean square of successive differences; PNN50 = percentage of adjacent cycles that are greater than 50 ms apart; HRV = Heart rate variability; SDNN = Standard deviation of all N-N intervals; RR = R to R intervals; SD = Standard deviation; E/R = Effort divided by reward; D/C = Demand divided by control; Mdn = Median; Sum = Sumscore; Tri = Triangulation of data; HL = Based on high low scores; HLC = Based on clinical high low scores; _l = measured during leisure time; _r = measured during rest; _w = measured during a workday; wln = measured during a period including workday, leisure time and night;_n = measured during a night; _t = Measured during a task; n.r. = Not reported; ne = No effect.

The second hypothesis states that there is a negative association between the reported parasympathetic outcomes and job stress/burnout. Reported outcomes on HF, RMSSD and PNN50 were included in this systematic review. The association between parasympathetic measures and job stress/burnout are also summarized in S2 Appendix. Thirty-four outcomes were reported on HF. Only 33 outcomes are reported in S2 Appendix as Hanson et al. [59] reported on a non-significant negative association for work leisure period, which was not a defined category in our study. Seven reported outcomes were significantly negative, two were significantly positive. Twenty-seven outcomes were reported on RMSSD, five of the reported outcomes were significantly negative, one was significantly positive. One study outcome was mixed on the conclusion; on the first day of the assessment no effect was found, whereas on the second day a significant and negative effect was found [51]. Six outcomes were reported on PNN50. Only one of the reported outcomes was significantly negative. One further remark has to be made for S2 Appendix. For PNN50, two of the outcomes were measured *in rest*. One of the outcomes was reported on a significant negative effect for the female sample [39] while the other reported outcome was positive, but non-significant for the male sample.

To summarize, we found 13 significantly negative reported outcomes for the parasympathetic outcomes, compared to 3 significantly positive reported outcomes. The relatively high number of negative effects seems to be in support of the second hypothesis, at least as far as the HF and RMSSD outcomes are concerned. However, most of the 67 reported outcomes were non-significant.

### 3.3 Burnout and job stress

The third hypothesis states that the association between burnout and HR(V)/EDA is stronger than the association between job stress and HR(V)/EDA. Six outcomes were reported on the HR-burnout association, however, only one was significantly positive [70], with the caution that the burnout group was entered as a dummy variable and part of a multiple regression analysis. Twenty-nine outcomes were reported on the HR-job stress association. Eleven outcomes were significantly positive. These results indicate the opposite of the hypothesis, the association between job stress and HR seems to be found more often than the association between burnout and HR.

As for the parasympathetic outcomes, seven outcomes were reported on the HF-burnout association. Only one reported outcome had a significant negative effect. Twenty-seven outcomes were reported on the HF-job stress association. Five reported outcomes were significantly negative. Five outcomes reported on the RMSSD-burnout association. Only one reported outcome had a significant negative effect. Twenty-two outcomes were reported on the RMSSD-job stress association. Four reported outcomes were significantly negative, one of the effects was mixed [51]. Note that there are two articles and 11 reported outcomes using both JDC and ERI to divide subjects in high/low strain. In sum, these results do not indicate that the parasympathetic association between burnout and HRV is found more often than the association between job stress and HRV.

### 3.4 Exploratory analysis

Because we were unable to perform a meta-analysis and for purposes of generating hypotheses, we explored whether the effects changed as a result of sample size, sex, or age (Table 3). In order to get a good contrast between samples, the results were analyzed by median splits based on these three variables. We expected HR to have a positive association with job stress and burnout. For the parasympathetic measures, a negative direction was expected. Interestingly, articles with higher sample sizes, and thus presumably providing more power to find a true effect, indeed found twice as much of the hypothesized effects than articles with lower sample sizes. The interpretation of the sex split is less clear as the samples are mixed, but the samples with a higher percentage of women seem to have more negative parasympathetic effects (11 vs 1). No age effects were found.

**Table 3:** Amount of positive (HR) and negative (parasympathetic) significant reported effects.

			HR (35)		Parasympathetic (67)
	*Median split*	*Range*	*# positive effects*	*Range*	*# negative effects*
**Sample size**	lower	20–95	4 (18)	17–135	4 (34)
	higher	100–1552	8 (17)	159–9924	9 (33)
**Sex proportion**	lower	0% females	7 (18)	0–12% females	1 (35)
	higher	8–100% females^a^	4 (16)	19–100% females ^b^	11 (30)
**Age**	lower	26.9–42.5	5 (17)	29.1–41.6	5 (30)
	higher	42.8–51.2	7 (17)	41.9–51.2^c^	5 (28)

The number of reported outcomes is in brackets. HR = heart rate. All reported outcomes were median split on sample size, sex or age.

^a^ A median split was performed on the basis of the percentage of females as in a general median split it would be arbitrary which of the female outcomes would be included in the higher % sample.

^b^ The median split was performed on the basis of the percentage of females. The sample was not exactly split in half because the median included a study with 6 reported outcomes. Therefore it would be arbitrary which of the reported outcomes would be included in the higher or lower percentage sample. We avoided this problem by including 30 outcomes in the higher % sample and 35 in the lower % sample.

^c^ The median split was performed on the basis of age. The sample was not exactly split in half because the median included a study with 6 reported outcomes. Therefore it would be arbitrary which of the reported outcomes would be included in the higher or lower age sample. By including 28 outcomes in the higher age sample (and 30 in the lower) we avoided this problem.

## 4. Discussion

This systematic review focuses on the relationship between job stress and burnout on the one hand and parasympathetic and sympathetic activity on the other hand. The current review could be considered as both a replication, update and an extension of previous work on this topic. The overall aim of this review was to better understand the association between job stress/burnout and HR(V)/EDA, which parameters might prove useful, and which recording periods are favorable over others to analyze.

### 4.1 Main findings for the hypotheses

The first hypothesis stated that there is a positive association between HR measures and job stress/burnout. First, support for this hypothesis came from both the high number of positive associations and the ratio of positive and negative effects (12 vs. 2), which clearly showed that the likelihood of a positive association between HR and job stress/burnout is higher. In other words, the results of this review support that high levels of job stress and burnout are associated with an increased HR. Second, one-third of all reported outcomes (12/35) showed a significant positive association between HR and job stress/burnout. However, if we leave burnout out of the analysis, 11 of 29 of the effects for job stress were positive. The number of articles on burnout included in this systematic review was too small to draw any firm conclusions. As van Doornen [69] also pointed out, the daily hassles of job stress may be incomparable with scales of exhaustion, depersonalization and personal accomplishment as a result of enduring job stress, but further research is necessary to investigate this claim.

Hypothesis 2 stated that there is a negative association between parasympathetic outcomes, indicated by HRV parameters, and job stress/burnout. Support for this hypothesis came from both the number of negative directions and the ratio of negative and positive effects (13 vs. 3), which showed that the likelihood of a negative association is higher. In addition to the ratio, one fifth (13/67) of all reported outcomes showed a significant negative association. If we leave burnout out of the analysis, one sixth (7/42) of the effects were negative. Twelve of the 19 articles included by Jarczok et al. [35] were also included in this systematic review. The twelve articles included in both systematic reviews showed comparable results, although Jarczok et al. [35] included fewer reported outcomes for the parasympathetic measures than in our study (38 articles). In Jarczoks’ sample, half of the parasympathetic outcomes were significantly negative (17/33) on the (sub)scales. Our analysis resulted in a significant negative effect in only one fifth (6/29) of the outcomes on the full scales, which explains the difference as the unit of analysis of the scales is different. For example, Jarczok et al. [35] also included effects of the separate scales for demands, control, effort or reward of the JDC and ERI (workplace stressors vs job stress) models while we only considered articles that reported results on the entire scale. However, the direction of the effect in both studies (i.e. the study by Jarczok et al. [35] and the current study) was overwhelmingly negative, that is, higher levels of job stress are associated with lower parasympathetic activation. It is worth mentioning that the correlation between HF and RMSSD is usually high (>.90) [43]. Therefore, it seems intuitive to expect a significant association of RMSSD if HF also has an effect, and vice versa, which results in an overestimation of the effects found in our study, due to multicollinearity. Finally, although SDNN is not a primarily sympathetic or parasympathetic outcome measure, there was a tendency towards a negative association between SDNN and job stress/burnout in our study.

The third hypothesis stated that there is a stronger association between psychophysiological measures and burnout than between psychophysiological measures and job stress. However, this hypothesized relationship could not be confirmed. One explanation for this might be the small number of included articles on burnout. Another potential reason might be that people with burnout symptoms do not experience job stress symptoms anymore as they are on sick leave which might have a calming effect on the body, and thus the (para)sympathetic measures.

In conclusion, it is important to state that most of the reported outcomes were non- significant, and for some articles that reported that there was no effect, we could not determine the direction of association. We did find partial support for the two hypotheses regarding a positive direction for the HR measures and a negative direction for the associations of parasympathetic measures with job stress and burnout. The ratio for the HR measures (12 vs. 2) was slightly higher than the ratio for the parasympathetic measures (13 vs. 3). Also, some evidence was found that articles with a larger sample size more often found a significant association. However, the third hypothesis regarding a stronger association between psychophysiological measures and burnout as opposed to job stress was not supported.

### 4.2 Methodological considerations

Related to the findings for the three hypotheses, four observations can be made regarding methodology and measurement. First, most reported HR outcomes came from rest (n = 11) measures. The number of reported parasympathetic outcomes was almost twice as high, most measures were taken at rest (n = 16), workday (n = 13), for 24 hours (n = 14), and at night (n = 13). It is remarkable that only few articles used rest measures since these seem to be most easily obtained, although some researchers may disagree. However, a rest protocol does assure that there is no movement or psychosocial demand on the participants, which might result in an artifact free signal. Also, there is some heterogeneity within these rest measures. Borchini et al. [40] pointed out that a strict standardized ECG protocol is necessary to obtain precise results. We recommend to include rest as a baseline measure in future studies. The baseline measure could also be used to adjust for between-person variation in psychophysiological recordings. It is worth noting that both HR and EDA show diurnal variation which suggests that this has to be taken into account as well when obtaining rest measures [53–55].

Second, regarding the prolonged measurements of HRV there are two points worth noting. First, Uusitalo et al. [51] suggest that nonlinear measures of HRV are less movement prone. In comparison with frequency domain measures, they argue that time domain and nonlinear HRV might prove to be more stable and suitable for situations in which a person may show a lot of movement resembling the real-life situations. Only few included articles report on controlling for physical activity, which is expected to have an influence. It is recommended to adjust for these movement artifacts if possible. In addition, Kamath et al. [81] mentioned the influence of respiration on the HF component of frequency domain measures; as breathing decreases, HF decreases as well. The authors state that HF measures can only be obtained in case one controls for breathing which might not be possible for all mobile devices that are currently on the market. We recommend careful consideration controlling for movement and breathing if possible.

Third, Clays et al. [43] point at the fact that one cannot compare all parameters obtained in different time intervals as they are dependent on time of analysis. For instance, SDNN is highly dependent on the length of the recording, and there are apparent differences in the duration of measurement intervals between studies. SDNN is typically used for 24-h recordings only. This is in line with remarks made by Kamath et al. [81] that long term measurements are preferably analyzed by time domain methods, and short term measures are preferably analyzed by frequency domain methods. With time domain measures it is difficult to discriminate between sympathetic and parasympathetic measures. In the current review, five of the outcomes on rest measures, which are usually 3–15 minutes long, were time domain measures as opposed to 11 outcomes on the frequency domain. There is a need for guidelines on the use of time domain or frequency domain methods and the duration of assessment from different laboratories [81]. Although a recommendation on duration of measurement is beyond the implications of this review, we would recommend reporting exact timeframes, methods of analysis, and transformations and filters applied to compare data more easily, even if mobile devices are not used in laboratories, but in real life situations.

Fourth, a final notion on real-life measurement is made by Rau et al., [46]. The authors suggest that the assessment of leisure time differs between studies, which may consequently lead to differences in findings. Some studies operationalize leisure time as the time between work and sleep while others consider resting days as leisure time. We recommend the use of after work leisure time as a separate terminology from a day that is completely without work.

Many of the reported outcomes are not independent. Therefore, we evaluated how many of the significant associations came from the same study. For the HR outcomes, the 12 significant positive outcomes came from 11 articles, whereas the 19 non-significant reported outcomes came from 14 articles (no significance test was reported for 3 outcomes; and 1 study reported mixed effects.) Thus, there appeared to be more independency among the significant associations than among the non-significant associations, which increases the reliability of the significant relationships. For the parasympathetic reported outcomes, the 13 significant negative outcomes came from 10 articles, whereas the 49 non-significant outcomes came from 22 articles (no significance test was reported for 2 outcomes; and the association for 3 outcomes was in the opposite direction of our hypothesis–positive instead of negative.) Again, there was more independency among the significant associations than among the non-significant associations.

Regarding the exploratory analyses it is worth noting that for sample size both sympathetic and parasympathetic measures show a tendency to obtain more associations in the hypothesized directions if the sample size is larger. A larger sample size often means a higher power, which implies a higher chance of obtaining a true effect, in this case a positive relationship between HR and a negative relationship between parasympathetic functioning and job stress/burnout which increases our trust in the relationships that we found. We do not want to extrapolate conclusions based on sex as we had less female samples, which is an indication that more female samples are needed in future studies to increase our confidence.

### 4.3 Directions for further research

A particular strength of the current study is that the results reported by Jarczok et al. [35] were replicated. This is especially relevant as psychological research is currently dealing with a replication crisis [82]. Second, this review extended the former one by only considering the full scales of job stress, adding the concept of burnout, and including EDA as a purely sympathetic marker. A third strength of this review is the additional search that was performed for gray literature by emailing all included authors. In spite of this strength, this study has a limitation on ‘vote counting’. If bivariate correlations or adjusted models with the same covariates are unavailable, it is difficult to summarize the true effect. The problem with vote counting is that it does not consider the magnitude of an effect, and control for heterogeneity or moderation is impossible. A second limitation is that we were unable to perform a meta-analysis which makes the results less compelling than they could have been.

We also have some additional recommendations. First, there is high variability in the number of covariates and bivariate or partial correlations reported. We strongly recommend to report both bivariate relations and adjusted models as this can seriously alter the effects that are found. This becomes even more evident as we consider that, as mentioned earlier, there is dependency between the reported outcome measures as they are all based on (variation in) HR, and some outcome measures have high correlations (i.e. the correlation between HF and RMSSD is usually high (>.90) [83]. We recommend that studies use the STROBE (Strengthening the Reporting of Observational Studies in Epidemiology; [84] statement to guide reporting for future studies.

Second, the use of validated questionnaires is highly recommended, there was an abundance of studies using only one job stress question making it unclear what the construct of the measure was (none of those non validated studies were included in this review). As the subjective meaning of job stress differs between jobs and people within these jobs, for comparability a thorough investigation is important.

Third, in burnout research there is often a predisposed clinical cut-off. In the job stress samples this is not always the case as some report on the quadrants while others use a median split or a 20/80 division. It is recommended to use the same validated cut-offs. A problem with comparability between articles arises as some articles included relatively healthy employees while others included a relatively large number of stressed individuals. In addition, providing the data along with the publication would allow for a meta-analysis of individual data.

Fourth, it is essential to report on both sex and age as there are few articles that report differences between men and women [38,39]. The concept of job stress might not be applicable to men and women alike as Riese [78] already mentioned. The original constructs might hold true more to men than to women. The psychophysiological profile of stress and burnout in women might be different.

Of course the question remains to what extent self-report questionnaire measures correspond to physiological measures of job stress. HRV is often used as a measure of stress while it does not always match the subjective stress that people experience. It would therefore be worth constructing a psychophysiological profile in which the within-subject baseline is taken into account, which would be possible with a baseline measurement. People differ from each other in baseline measures. It is unclear if their baseline is the same in all circumstances, let alone if the baseline can change in situations where they get stressed. In other words, people might have a moving baseline. However, the vast majority of research until now has been cross-sectional in nature, and comparisons were made between subjects or between groups. The baseline of the subjects is only considered in some of the more recent research [80].

The fact that there is a small difference in the number of reported parasympathetic associations between our study and the study by Jarczok et al. [35] stresses the importance of reporting all bivariate correlations or means on all scales, for this will enable a meta-analysis, as was also suggested in more recent research [85]. Moderation analysis can then be performed on different sets of confounders and covariates. The current systematic review includes articles that use a variety of confounding adjustments and covariates, which makes it difficult to compare them.

Lastly, no EDA studies were found that met our criteria. A few studies were considered for inclusion. For instance, Cendales-Ayala et al. [86] did a simulation study in which it was shown that high demands in bus drivers resulted in significantly increased EDA. Considering the sympathetic nature of the EDA, we expected to find more studies. However, only recently it is possible to obtain EDA measures via mobile devices, such as wristbands, in real life during prolonged periods of time. Based on this development we expect to find more studies on EDA and job stress in the near future.

### 4.4 Conclusion

In conclusion, this study examined whether HR was positively associated with job stress and burnout. In addition, it examined whether there was a negative association between parasympathetic markers, and job stress and burnout. Support was found for both hypotheses. No support was found for the hypothesis that the association with burnout was stronger than it was for job stress. There is a need for more extensive reporting of effect directions, and on female samples, which were underrepresented in the current review. In this sample job stress was mostly related to increases in HR and decreases in RMSSD, HF and SDNN. Maybe these measures can be used as indicators and warning signals of increases in job stress, whereas the relationship with burnout is less clear.

## Supporting information

S1 Appendix Searchterms. (DOCX)

S2 Appendix Association between HR/SDNN/HF/RMSSD/PNN50 and job stress/burnout by recording period. (XLSX)

S3 Appendix Newcastle- Ottowa Risk of Bias table for all studies. (DOCX)

S4 Appendix PRISMA checklist. (DOCX)

S5 Appendix PROSPERO protocol. (DOCX)

S6 Appendix Raw_Codesheet risk of bias. (XLSX)
