# Supplementary material for: Associations of sympathetic and parasympathetic activity in job stress and burnout: A systematic review
Source: PLoS One. 2018 Oct 18;13(10):e0205741. doi: 10.1371/journal.pone.0205741 (PMC6193670; doi:10.1371/journal.pone.0205741)
Supplement: S1 Appendix — (DOCX) [file pone.0205741.s001.docx]

**Appendix A**

All searches were conducted on the 23^rd^ of December 2016.

Here is a link to the databases:

Medline and Pubmed:

[**https://www.ncbi.nlm.nih.gov/pubmed/**](https://www.ncbi.nlm.nih.gov/pubmed/)

Web of Science:

[**www.webofknowledge.com/**](http://www.webofknowledge.com/)

PsycInfo:

[**https://www.ebsco.com/products/research-databases/psycinfo**](https://www.ebsco.com/products/research-databases/psycinfo)

Medline*

| *1* | Heart Rate/ or (cardiac rate* or heartbeat or heart beat or interbeat or heart rhythm or heart rate* or rate* pulse* or cardiac chronotrop*).mp. |
| --- | --- |
| 2 | galvanic skin response/ or psychophysiology/ or (GSR or electrophysiology or skin potential or galvanic or electrodermal or psychogalvanic or psychophysiology).mp. or ((skin or dermal) adj3 (electric* or conduct* or resistance or physiology)).mp. |
| 3 | burnout, professional/ or (((work or job or occupation*) adj3 Stress) or burnout or ((work or job or occupation*) adj3 exhaustion) or ((work or job or occupation*) adj3 strain)).mp. |
| 4 | 1 or 2 |
| 5 | 3 and 4 |
| 6 | limit 5 to (english language and yr="2000 -Current") |

**the .mp keyword is used to search in multi-purpose (mp) fields. It usually includes fields like Title, Abstract and Subject heading.*

Web of Science

| *# 6* | *(#2 AND #4) AND* ***LANGUAGE:*** *(English)*  *Indexes=SCI-EXPANDED, SSCI, A&HCI, ESCI Timespan=2000-2016* |
| --- | --- |
| # 5 | #2 AND #4  Indexes=SCI-EXPANDED, SSCI, A&HCI, ESCI Timespan=All years |
| # 4 | #1 OR #3  Indexes=SCI-EXPANDED, SSCI, A&HCI, ESCI Timespan=All years |
| # 3 | TS=((heart rate*) OR (cardiovascular reactivity) OR (psychophysiology) OR (cardiac) OR (heartbeat*) OR (heart beat*) OR (interbeat) OR (heart rhythm) OR (rate* pulse*) OR (physiology))  Indexes=SCI-EXPANDED, SSCI, A&HCI, ESCI Timespan=All years |
| # 2 | TS= ((((work or job or occupation*) NEAR/3 Stress)) OR (burnout) OR (((job or occupation*) NEAR/3 exhaustion)))  Indexes=SCI-EXPANDED, SSCI, A&HCI, ESCI Timespan=All years |
| # 1 | TS=(GSR OR Electrophysiology OR galvanic OR Electrodermal OR psychogalvanic OR psychophysiology OR ((skin or dermal) NEAR/3 (galvanic OR electr* OR conduct* OR resist* OR potential OR physiology)))  Indexes=SCI-EXPANDED, SSCI, A&HCI, ESCI Timespan=All years |

PsycInfo

| *1* | *heart rate/ or cardiovascular reactivity/ or physiological arousal/ or physiological correlates/ or psychophysiology/ or stress reactions/ or (cardiac rate* or heartbeat or heart beat or interbeat or heart rhythm* or heart rate* or rate* pulse* or cardiac chronotrop*).mp.* |
| --- | --- |
| 2 | galvanic skin response/ or skin resistance/ or basal skin resistance/ or exp skin electrical properties/ or skin potential/ or psychophysiology/ or (GSR or electrophysiology or skin potential or galvanic or electrodermal or psychogalvanic or psychophysiology).mp. or ((skin or dermal) adj3 (electric* or conduct* or resistance or physiology)).mp. |
| 3 | occupational stress/ or (burnout* or burn-out* or ((work or job or occupation*) adj3 stress*) or ((work or job or occupation*) adj3 exhaustion) or ((work or job or occupation*) adj3 strain*)).mp. |
| 4 | 1 or 2 |
| 5 | 3 and 4 |
| 6 | limit 5 to (english language and yr="2000 -Current") |

Embase

| *1* | *exp "heart rate and rhythm"/ or (cardiac rate* or heartbeat or heart beat or interbeat or heart rhythm or heart rate* or rate* pulse* or pulse rate* or cardiac chronotrop*).mp.* |
| --- | --- |
| 2 | electrodermal response/ or (GSR or electrophysiology or skin potential or ((skin or dermal) adj3 (galvanic or electrodermal or electric* or conduct* or psychogalvanic or resistance or psychophysiology or physiology))).mp. |
| 3 | burnout, professional/ or job stress/ or (((work or job or occupation*) adj3 Stress) or burnout or ((work or job or occupation*) adj3 exhaustion) or ((work or job or occupation*) adj3 strain)).mp. |
| 4 | 1 or 2 |
| 5 | 3 and 4 |
| 6 | limit 5 to (english language and yr="2000 -Current") |
