# Supplementary material for: Associations of sympathetic and parasympathetic activity in job stress and burnout: A systematic review
Source: PLoS One. 2018 Oct 18;13(10):e0205741. doi: 10.1371/journal.pone.0205741 (PMC6193670; doi:10.1371/journal.pone.0205741)
Supplement: S3 Appendix — (DOCX) [file pone.0205741.s003.docx]

**Appendix C. Summary of the Newcastle-Ottowa Risk of bias scores**

| Study | Risk of bias score (Higher means less bias) |
| --- | --- |
| Borchini (2015)[40] | 7 |
| Clays (2011)[27] | 7 |
| Collins (2005)[53] | 7 |
| Doornen (2009)[54] | 7 |
| Ekstedt (2004)[42] | 7 |
| Eller (2011)[46] | 7 |
| Eriksson (2016)[55] | 7 |
| Fauvel (2001)[56] | 7 |
| Hanson (2001)[41] | 7 |
| Hernández-Gaytan (2013)[59] | 7 |
| Herr (2015)[60] | 7 |
| Hintsanen (2007)[28] | 7 |
| Jarczok (2016)[61] | 7 |
| Johnston (2016)[62] | 7 |
| Karhula (2014)[64] | 7 |
| Lee (2010)[66] | 7 |
| Lennartsson (2016)[67] | 7 |
| Loerbroks (2010)[18] | 7 |
| Moya-Albiol (2010)[39] | 7 |
| Nomura (2005)[68] | 7 |
| Riese (2004)[47] | 7 |
| Uusitalo (2011)[37] | 7 |
| vanAmelsfoort (2000)[71] | 7 |
| Vrijkotte (2000)[9] | 7 |
| Chandola (2008)[52] | 6 |
| Hamer (2006)[57] | 6 |
| Jönsson (2015)[3] | 6 |
| Kang (2004)[63] | 6 |
| Rau (2001)[44] | 6 |
| Teisala (2014)[48] | 6 |
| Henning (2014)[58] | 5 |
| Kotov (2012)[65] | 5 |
| Ohira (2011)[69] | 5 |
| Poorabdian (2013)[38] | 5 |
| Bishop (2003)[50] | 4 |
| Morgan (2002)[20] | 4 |
| Butterbaugh (2003)[51] | 2 |
| Salavecz (2010)[70] | 1 |

The scale can be found at: <http://www.ohri.ca/programs/clinical_epidemiology/oxford.asp>

The Newcastle-Ottowa Risk of Bias scores are calculated for both cohort or case control studies. The coding manual has 7 questions related to ‘Selection’, ‘Comparability’ and ‘Exposure’.
