# Supplementary material for: Associations of sympathetic and parasympathetic activity in job stress and burnout: A systematic review
Source: PLoS One. 2018 Oct 18;13(10):e0205741. doi: 10.1371/journal.pone.0205741 (PMC6193670; doi:10.1371/journal.pone.0205741)
Supplement: S5 Appendix — (DOCX) [file pone.0205741.s005.docx]

Appendix E. PROSPERO protocol

Registration number: CRD42016035918

You can find the record over here: (<http://www.crd.york.ac.uk/prospero/display_record.php?ID=CRD42016035918>)

*PRISMA-P (Preferred Reporting Items for Systematic review and Meta-Analysis Protocols) 2015 checklist: recommended items to address in a systematic review protocol* Section and topic Item No Checklist item ADMINISTRATIVE INFORMATION Title:*

***Identification***

*1a Identify the report as a protocol of a systematic review*

Systematic review protocol for a systematic review into electrodermal activity (EDA) and heart rate (HR) for prediction of job related stress or burnout in the employed population.

***Update***

*1b If the protocol is for an update of a previous systematic review, identify as such*

It does not concern an update of a previous systematic review or meta-analysis

***Registration***

*2 If registered, provide the name of the registry (such as PROSPERO) and registration number*

The review protocol will be registered at PROSPERO.

***Authors***

*3a Provide name, institutional affiliation, e-mail address of all protocol authors; provide physical mailing address of corresponding author*

Corresponding author

Peter de Looff, p.de.looff@altrecht.nl

Protocol authors

Peter de Looff MSc^1,5^

p.de.looff@altrecht.nl

Henk Nijman PhD^1,5^

h.nijman@altrecht.nl

Robert Didden PhD^1,3^

r.didden@pwo.ru.nl

Petri Embregts PhD^2,4,6^

p.j.c.m.embregts@uvt.nl

1. Behavioural Science Institute, Radboud University, Montessorilaan 3, P.O. Box 9014, 6525 HR Nijmegen, The Netherlands
2. Tranzo, Tilburg School of Social and Behavioral Sciences, Tilburg University, P.O. Box 90153, 5000 LE Tilburg
3. Trajectum, P.O. Box 40012, 8004 DA Zwolle
4. Dichterbij Innovation and Science, Gennep, The Netherlands
5. Wier, Specialized and Forensic Care, Altrecht Aventurijn, Eikenpage 2, 3734 AC Den Dolder
6. Department of Medical and Clinical Psychology, Tilburg School of Social and Behavioral Sciences, Tilburg University, Tilburg, The Netherlands

***Contributions***

*3b Describe contributions of protocol authors and identify the guarantor of the review*

Peter de Looff is the guarantor. All authors contribute to the protocol planning, search strategy, assessment of the criteria for inclusion, and will give feedback on the manuscript.

***Amendments***

*4 If the protocol represents an amendment of a previously completed or published protocol, identify as such and list changes; otherwise, state plan for documenting important protocol amendments*

We will update the protocol in PROSPERO if a protocol amendment is necessary.

***Support: Sources***

*5a Indicate sources of financial or other support for the review Sponsor*

*5b Provide name for the review funder and/or sponsor Role of sponsor or funder*

Financial and other support for this systematic review (SR) is available through a research grant from “De Borg” and “DForZo”(Directie Forensische Zorg; Board of Forensic Care). De Borg is a collaboration of four mental health organizations for the clinical treatment of people with behavior disorders and mild to borderline intellectual disabilities (MBID). DForZo is part of “DJI” (Dienst Justitiele Inrichtingen; Ministry of Justice) and is responsible for executing detention sentences on people that are sentenced by the justice system. The SR is part of the dissertation by Peter de Looff, who also is the corresponding author.

*5c Describe roles of funder(s), sponsor(s), and/or institution(s), if any, in developing the protocol*

The funders have no role in developing the protocol for the SR. De Borg and DForZo are the funders of the project.

*INTRODUCTION*

***Rationale***

*6 Describe the rationale for the review in the context of what is already known*

In the first half of 2014, job related stress costed the Dutch economy over 800 million euro’s (ArboNed, 2014). Prolonged exposure to job related stress and inadequate coping can result in burnout (Swider & Zimmerman, 2010), while early detection of high stress levels might aid prevention and increase the opportunities for social support between colleagues, which is known to be an important factor in the prevention of burnout (Rose, 1999; Taris, Houtman, & Schaufeli, 2013). Two psychophysiological measures, i.e. electrodermal activity (EDA) and heart rate (HR), might aid in the detection of high stress levels. This review investigates the relationship between these measures and job related stress and burnout. It will contribute to our understanding of (the prevention of) high stress levels and burnout. Burnout also has a major personal, organizational, and societal impact.

To our knowledge, the relationship between job related stress, burnout and psychophysiological measures has not been investigated in MBID-samples, so we expand our search criteria to the general employed population.

***Objectives***

*7 Provide an explicit statement of the question(s) the review will address with reference to participants, interventions, comparators, and outcomes (PICO)*

Aim of this review is to provide a critical analysis of results of studies on the association between the psychophysiological measures and job related stress and burnout among the employed population. Consequently, our aim is to explore what is known about EDA and HR (and some measures of heart rate variability) as predictive variables in relation to job related stress and burnout.

*METHODS*

***Eligibility criteria***

*8 Specify the study characteristics (such as PICO, study design, setting, time frame) and report characteristics (such as years considered, language, publication status) to be used as criteria for eligibility for the review*

*Study designs quality criteria*

Both quantitative and qualitative studies may be included in this SR. Quantitative studies are both experimental and observational in nature. Qualitative studies do not evaluate quantitative data, but can be used to gain greater insight in the possible association between variables. Two reasons can be given for including different types of designs. First, for quantitative studies both experimental and observational designs will be included as we want to explore a possible relation between psychophysiology and job related stress or burnout. Second, as Petticrew and Roberts (2006) point out: “Systematic reviewing is, or has the potential to be, a broad church and can deal with a variety of questions, study designs, and methods within the same review”. The different designs are used to answer different questions (Petticrew & Roberts, 2006). If one wants to know what works, randomized controlled trials are the preferred design. However, if one wants to know how it works, qualitative designs are more appropriate. Since our research question does not specifically focus on effectiveness, salience, safety, acceptability, satisfaction or appropriateness of an intervention or process, we include both types of designs to get a broad perspective on the association of job related stress, burnout and psychophysiological measures.

There are a number of possible taxonomies to consider. We chose a taxonomy established by Pettigrew and Roberts (Petticrew & Roberts, 2006). Pettigrew and Roberts suggest to use ‘typologies’ instead of ‘hierarchies of evidence’. Five categories (typologies) can be distinguished: (1) Randomized controlled trials (RCT, most appropriate to study effectiveness, what works?); (2) Quasi experimental (control group but not random); (3) Uncontrolled studies (no control group); (4) Studies assessing etiological relationships: Prospective cohort studies and retrospective case control; (5) Qualitative studies (For instance, How does it work?).

The taxonomy serves as a means to asses studies on relative strength and weight of effectiveness (Larkin, Jahoda, & MacMahon, 2013; Petticrew & Roberts, 2006). Although this is not a specific aim of the review it serves as a tool for categorizing the included studies. This does not imply that studies with different designs will be compared. That is, observational studies will be compared to observational studies, and they will not be compared to experimental studies. However, it does imply that different types of designs will be included in the review, and they will be compared within each category.

In sum, studies will be designated to one of five categories:

| Comparison designs | | | **Random** | **Control-group** | **Stimulus/Intervention** |
| --- | --- | --- | --- | --- | --- |
| Experimental | **1** | RCT | Yes | Yes | Yes |
|  | **2** | Quasi |  | Yes | Yes |
|  | **3** | Uncontrolled |  |  | Yes |
| Observational | **4** | a.Cohort |  |  |  |
|  |  | b.Case-control |  | Yes |  |
| Qualitative | **5** | Qualitative |  |  |  |

**Table 1. Studies will be categorized using the taxonomy matrix.**

***Inclusion criteria***

From Lorber (2004)

Studies examine the relationship between psychophysiology and job related stress or burnout.

Participants

The studies contain samples of the adult human employed population.

Interventions

In line with Lorber (2004), we include studies that have a baseline measure of HR(V) or EDA. We will tabulate these baseline measures in relation to job related stress and burnout if possible. In addition, also studies including some kind of experimental task that participants (or groups) had to perform were formed in retrospect based on a job related stress or burnout measure. We will also include the latter if they provide data on the psychophysiological measures. Thus, studies will have to include some measure of HR(V) or EDA measured during baseline or an experimental task and an assessment of job related stress or burnout.

Comparators

Comparisons will be made if different groups with different levels of job stress or burnout were considered. A concern with this approach is comparability across studies as we expect heterogeneity between studies with different designs (Egger, Schneider, & Smith, 1998). The assessment of experimental studies relies on the calculation of effect sizes. Egger, Schneider and Smith (1998) suggest to do the same with observational studies, but less prominent. For observational studies the assessment of the sources of heterogeneity are of greater importance, and we intend to follow this suggestion. Studies will have to include sufficient data to allow calculation of effect sizes, and a test statistic or p- value for a test of interest though.

Outcomes

Outcomes will include stress measures. More specifically, studies will be included if they report on outcomes of job related stress or burnout. The EDA measures preferably consist of level, amplitude or frequency, the HR measure of baseline HR, HR reactivity, or HRV.

Timing of follow up

There will be no restrictions on the timing of follow up.

Setting

There will be no restrictions on the type of setting.

Language

Only English articles will be eligible for the review. We prefer the article format, if a study is available both as an article and a dissertation.

Exclusion criteria

Samples with students, animals.

Articles written in another language than English.

***Information sources***

*9 Describe all intended information sources (such as electronic databases, contact with study authors, trial registers or other grey literature sources) with planned dates of coverage*

*Search strategy*

We intend to use a similar search strategy as Lorber (2004).

Search strategy:

1. Database search. Psychinfo, Medline, Dissertation abstracts international. We add: ISI Web of Science and Embase

2. Reference lists of retrieved articles and review articles

3. We excluded: Contact authors

4. We excluded: Post queries through relevant media (The society for psychophysiological research)

The reason for exclusion of “contact authors” is a response bias. Some, but not all, authors will respond. The same argument can be considered for “posting queries through relevant media”.

*10 Present draft of search strategy to be used for at least one electronic database, including planned limits, such that it could be repeated*

The following search strings will be used in combination. The wording for the first two terms were extracted from Lorber (2004).

1. electrodermal, skin conductance, skin potential, galvanic, physiology, and psychophysiology
2. interbeat, heart rate, cardiac (addition “heart rate variability”)
3. burnout, job stress, job burnout, job exhaustion, occupational stress, occupational burnout, occupational exhaustion

***Study records***

*11a Data management. Describe the mechanism(s) that will be used to manage records and data throughout the review*

We will use Covidence to assess eligibility of the studies. We will screen all studies/articles based on the inclusion and exclusion criteria. We will explain the software to reviewers that are not familiar with the software. For the calculation of the effect sizes and management of tables we will use Review Manager. The latter is software developed by the Cochrane Collaboration to conduct systematic reviews.

*11b State the process that will be used for selecting studies (such as two independent reviewers) through each phase of the review (that is, screening, eligibility and inclusion in meta-analysis)*

The lead author will conduct a search together with a librarian. The lead author will review articles, decide on their eligibility, and decide on inclusion of the articles based on the inclusion criteria. A study (Edwards et al., 2002) showed that the use of two reviewers to screen articles on eligibility could increase the number of relevant articles by an average of 9%, so it is recommended to use multiple reviewers. For this reason, the other authors will review a subset of the articles that need to be screened in order to establish interrater reliability. We will record the reason for excluding articles. In a final session we will discuss the reasons for in- or excluding articles. All relevant steps in the process will be registered.

*11c Describe planned method of extracting data from reports (such as piloting forms, done independently, in duplicate), any processes for obtaining and confirming data from investigators*

In order to explore if HR(V) and EDA are considered reliable predictors of job related stress or burnout we will summarize these findings in a table. We will also note the parameters extracted from the EDA or HR(V) signal and the questionnaires that were used to establish job related stress and burnout. Each category of the study design taxonomy will be tabulated separately as can be seen in table 1.

***Data items***

*12 List and define all variables for which data will be sought (such as PICO items, funding sources), any pre-planned data assumptions and simplifications*

We will extract the age and gender of the participants. No specific interventions will be extracted. Comparisons will be made if they occur naturally as a between group difference in job stress or burnout. If there are different groups to compare they will consist of naturally occurring groups containing people with low vs high scores on a job stress related or burnout scale in observational studies. For experimental studies these groups already exist. The outcomes that will be registered consist of job related stress or burnout. We will report mean or percentile scores whenever possible. For the outcome measures on EDA we will report the level, amplitude or frequency. Different parameters can be extracted from the EDA signal and we will follow the taxonomy made by Boucsein (Boucsein, 2012). For HR we will report on time or frequency domain methods as reported by the taskforce of the European Society of Cardiology the North American Society of Pacing Electrophysiology (Electrophysiology, 1996). According to a recent meta-analysis (Brindle, Ginty, Phillips, & Carroll, 2014), most important in relation to HR reactivity and psychological stress seems to be root mean square of successive differences (RMSSD) and the correlation dimension (D2).

***Outcomes and prioritization***

*13 List and define all outcomes for which data will be sought, including prioritization of main and additional outcomes, with rationale Risk of bias in individual studies*

Our main outcome will focus on the differences in EDA and HR(V) for people with and without job related stress or burnout. To determine the level of job related stress of burnout we will use the measures used by the researchers. For example, the Dutch version of the Maslach Burnout Inventory (Maslach & Jackson, 1981), contains a 5-point norm scale ranging from ‘very low’ to ‘very high’ on this measure of burnout. If the outcomes are reported we can include these in the tabulation to distinguish between low and high levels of burnout.

*14 Describe anticipated methods for assessing risk of bias of individual studies, including whether this will be done at the outcome or study level, or both; state how this information will be used in data synthesis*

In RCT designs it is common to include a measure of bias based on a form with the most typical sources of bias. An example of such a form is the Cochrane Collaboration tool for assessing the risk of bias. It is expected that the majority of studies to be included in this SR are observational in nature, and have bias at the highest possible level. For this reason, an additional tool exists from the Cochrane Collaboration on assessing the risk of bias in Cohort studies. Both the study and outcome level will be assessed.

***Data synthesis***

*15a Describe criteria under which study data will be quantitatively synthesized*

We will be able to disseminate parameters from the studies. Depending on the design of the study we will be able to quantify and compare them between studies. In some cases studies are expected to be heterogeneous on outcome measures. We will try to tabulate data as much as possible. We will include study characteristics of the population, methods and results, and we will use Rev Manager to assess these studies.

*15b If data are appropriate for quantitative synthesis, describe planned summary measures, methods of handling data and methods of combining data from studies, including any planned exploration of consistency (such as I2 , Kendall’s τ)*

*15c Describe any proposed additional analyses (such as sensitivity or subgroup analyses, meta-regression)*

*15d If quantitative synthesis is not appropriate, describe the type of summary planned*

We will use the guidelines for a narrative review formulated by the Centre for Reviews and Dissemination (2009) in case qualitative studies are assessed.

***Meta-bias(es)***

*16 Specify any planned assessment of meta-bias(es) (such as publication bias across studies, selective reporting within studies)*

***Confidence in cumulative evidence***

*17 Describe how the strength of the body of evidence will be assessed (such as GRADE)*

The Grading of Recommendations Assessment, Development and Evaluation working group methodology (GRADE) will be used to evaluate the quality of the quantitative studies. All eligible quantitative studies will be entered into Review Manager to assess the quality of evidence ranging from high to very low. Evidence is considered of high quality if it is expected that further research will probably not change the effect. On the other hand, very low quality evidence suggests that further research may change the effect measure (Shamseer et al., 2015).
